# Animal and In Vitro Models as Powerful Tools to Decipher the Effects of Enteric Pathogens on the Human Gut Microbiota

**DOI:** 10.3390/microorganisms12010067

**Published:** 2023-12-29

**Authors:** Marco Calvigioni, Diletta Mazzantini, Francesco Celandroni, Emilia Ghelardi

**Affiliations:** Department of Translational Research and New Technologies in Medicine and Surgery, University of Pisa, 56127 Pisa, Italy; marco.calvigioni@med.unipi.it (M.C.);

**Keywords:** gut microbiota, intestinal infection, enteric pathogens, *Clostridioides difficile*, *Campylobacter jejuni*, *Escherichia coli*, *Salmonella enterica*, *Shigella*, *Vibrio cholerae*, *Bacillus cereus*

## Abstract

Examining the interplay between intestinal pathogens and the gut microbiota is crucial to fully comprehend the pathogenic role of enteropathogens and their broader impact on human health. Valid alternatives to human studies have been introduced in laboratory practice to evaluate the effects of infectious agents on the gut microbiota, thereby exploring their translational implications in intestinal functionality and overall health. Different animal species are currently used as valuable models for intestinal infections. In addition, considering the recent advances in bioengineering, futuristic in vitro models resembling the intestinal environment are also available for this purpose. In this review, the impact of the main human enteropathogens (i.e., *Clostridioides difficile*, *Campylobacter jejuni*, diarrheagenic *Escherichia coli*, non-typhoidal *Salmonella enterica*, *Shigella flexneri* and *Shigella sonnei*, *Vibrio cholerae*, and *Bacillus cereus*) on intestinal microbial communities is summarized, with specific emphasis on results derived from investigations employing animal and in vitro models.

## 1. Introduction

The gut microbiota is the most complex and biodiverse microbial consortium residing in the human host [1]. Bacteria in the human colon, the most populated section of the digestive tract, reach 10^12^ cells per gram of intestinal content, including more than 1500 facultative/obligate anaerobic species [2]. In healthy adults, *Bacillota* and *Bacteroidota* account for up to 90% of the total bacterial load, with *Bacteroides*, *Blautia*, *Clostridium*, *Enterococcus*, *Eubacterium*, *Faecalibacterium*, *Lactobacillus*, *Peptostreptococcus*, *Prevotella*, *Roseburia*, and *Ruminococcus* as the main representative genera [2,3]. *Actinomycetota* (i.e., *Bifidobacterium*), *Pseudomonadota* (i.e., *Escherichia*, *Klebsiella*), *Verrucomicrobiota* (i.e., *Akkermansia*), and other phyla represent a minority in the consortium [2,3]. In recent decades, efforts have been made to clarify the biophysiological roles of the gut microbiota, unravel its intricate microecology, and explore the numberless interactions occurring among gut microbes, host cells, and exogenous factors.

Since co-evolving in a mutualistic relationship with humans for thousands of years [4], the gut microbiota gradually acquired essential functions to ensure the correct homeostasis of the intestine and other organs and regions of the human body, thus playing crucial roles both locally and systemically [5]. In addition to various metabolic, trophic, and modulatory functions, the mere presence of microbial populations in the gut also reduces the rate of intestinal colonization by pathogenic microorganisms, further highlighting the primary importance of the gut microbiota in maintaining host health [5]. In fact, commensal microbes activate different mechanisms to counteract colonization, overgrowth, and invasion by enteric pathogens, carrying out defense strategies known under the name of “colonization resistance”. The production of killing/inhibitory compounds (e.g., bacteriocins, short-chain fatty acids (SCFAs)), harboring of strain-specific bacteriophages, and competition for nutrients (e.g., sugars, amino acids, iron, succinate) and/or adhesion sites (e.g., mucosal glycans) are the main mechanisms of colonization resistance due to the gut microbiota [6,7,8]. The loss of such a protective function, as in the case of radical qualitative and quantitative alterations in the gut microbiota following prolonged antibiotic therapies [9], increases vulnerability to endogenous (e.g., *Clostridioides difficile*) [10] and exogenous bacteria, as well as fungi (e.g., *Candida albicans*) [11,12]. Nevertheless, bacterial pathogens such as *Campylobacter jejuni*, diarrheagenic *Escherichia coli*, *Salmonella enterica*, *Shigella* spp., *Vibrio cholerae*, and *Bacillus cereus* can cause infection even in the presence of a healthy microbiota since their virulence is not always restrained by the local microbiota. In addition to the well-known pathogenetic mechanisms of each enteropathogen targeting human cells and tissues, the introduction of such microorganisms in the intestinal environment could also lead to aberrations in the gut microbiota itself. Fully understanding this side effect is important for human health, as it may contribute to the overall clinical manifestation and amplify the negative impact on the host by affecting the normal balance and functionality of the gut microbiota.

This review explores the effects of the most relevant bacterial intestinal pathogens on the gut microbiota, especially focusing on the results obtained from studies involving animal and in vitro models. Furthermore, the mechanisms underlying alterations in the intestinal microbial communities during infection and the colonization resistance to pathogens (if known) will be highlighted.

## 2. Animal Models or In Vitro Models for Gut Microbiota Research? A Controversial Decision

Since studies on humans are often limited by compliance, ethical issues, and the impossibility of constant monitoring and sample collection, many alternatives to clinical trials have been developed and introduced in common practice to study the gut microbiota and the effects of specific factors on its composition and functions.

Animal models were first proposed as human surrogates, especially to establish the association between certain clinical phenotypes and the harboring of distinctive intestinal consortia [13]. Various genetically engineered and drug-treated animal models were created to be used as disease models of specific pathological or infectious conditions [14]. Germ-free animals were also used as pristine canvases in evaluating the colonization process in uncontaminated body regions that are normally densely populated in naturally colonized animals [15,16]. Among the species available for experimental purposes, mice and rats have been commonly selected due to the similarity of their gastrointestinal tract and gut microbiota to those of humans [17,18]. Other non-rodent models have also been proposed, including zebrafish, rabbit, chicken, dog, and pig, even if often less relevant than rodents in the context of the gut microbiota [19]. Animal models are very flexible and able to represent several clinical pictures. Their user-friendly nature facilitates easy handling and administration of factors, along with the collection of samples that would otherwise be impossible to obtain from humans. Nevertheless, despite the efforts in developing such valuable models and the important discoveries made through their use, time and cost, low reproducibility, the difficulty in translating results to humans due to genetic, physiological, and dietary differences from animals, and the ethical issues related to the management of animals remain insurmountable limitations of using animal models [14].

In parallel to animal models, in vitro models mimicking as much as possible in vivo intestinal topography and conditions (e.g., oxygen partial pressure and gradient, pH, flow, nutrients, hepatopancreas-secreted fluids) have been designed and made available in recent years. The growing interest in these models arises from the need to circumvent the main limitations of working with animals and humans. In fact, working in vitro offers significant advantages, including high experimental reproducibility, continuous monitoring of culture conditions, convenient accessibility, cost-effectiveness, and avoidance of ethical concerns [20]. However, these advantages are attained by creating a working environment that is comparatively less intricate than the complex intestinal setting.

Research in this field began with traditional cell cultures in culture plates, which certainly represent a static environment that is far from the dynamics of the intestinal tract. The in vitro cultivation of a cell monolayer to test with intestinal microbes is easy to obtain, inexpensive, reproducible, and provides raw results for preliminary studies. Caco-2, HT-29-MTX, T84, LS174T, and CCD 841 CoN are the most used cell lines for gastrointestinal in vitro models due to their specific properties [21]. For instance, Caco-2 and HT-29 cells, the latter able to secrete mucus, were co-cultured in the presence of enterotoxigenic *E. coli* (ETEC) H10407 to investigate the role of mucus in inducing and shaping ETEC gene expression [22]. The same cell lines were also used to test the effect of conditioned media and microbial by-products from in vitro-cultured microbiota on the human host mimicking intestinal inflammation and cell immunomodulation [23]. In addition to the simplest two-dimensional (2D) cell monolayers, different gels and scaffolds can be used to support cell growth, offering a three-dimensional (3D) environment for cultivation. This approach more accurately represents the complex non-flat architecture of the intestinal mucosal environment [24,25]. Caco-2 and HT-29 cells were cultured on hydrogel scaffolds to demonstrate that mucus was a protective factor against bacterial colonization by adherent-invasive *E. coli* (AIEC) [26]. Raw and mucin-coated 3D gelatin electrospun membranes were successfully used as scaffolds to culture the human gut microbiota in vitro and reproduce the three-dimensional architecture of the intestinal mucosa and the arrangement of bacteria on it [27,28,29]. Cells assembled in organoids were also recently used in gut microbiota research. Organoids are spherical 3D culture systems derived from self-organized pluripotent or adult stem cells that can differentiate into lineages of intestinal epithelial cells and produce villus- and crypt-like structures resembling the architecture of the intestinal epithelium [30]. Single microbial species, pools of different microorganisms, or even fecal samples can be microinjected inside the lumen of organoids, making organoids effective in co-culturing epithelial cells with gut microbes [31]. In addition to in vitro models consisting of cells and microbes, increasingly technological devices have been developed to approach the complexity of the human intestine. Among these, Transwell, Human Oxygen Bacteria Anaerobic (HoxBan), Host–Microbiota Interaction (HMI), TNO In vitro Model (TIM-1), TNO In vitro Model of the Colon (TIM-2), Human–Microbiota Crosstalk (HuMiX), and Simulator of Human Intestinal Microbial Ecosystem (SHIME) are just some of the systems created in the last decades following advances in the fields of bioengineering, millifluidics, and microfluidics [21]. Modern artificial devices are currently being used in numerous in vitro studies focused on the gut microbiota. These devices recreate environments that faithfully replicate those found in vivo and deliver highly reproducible and translatable results [21].

To date, there is no right choice regarding how to conduct a scientific study on the gut microbiota. Each model displays its own pros and cons and the choice of one over the other often depends on the study objective and the degree of compromise investigators are willing to accept for their results.

## 3. Intestinal Pathogens and Gut Microbiota

This section dissects the impact of *C. difficile*, *C. jejuni*, diarrheagenic *E. coli*, *S. enterica* serovars Typhimurium and Enteritidis, *S. flexneri* and *S. sonnei*, *V. cholerae*, and *B. cereus* on the gut microbiota, examining alterations in its composition and in microbial-derived metabolites. In addition, it explores the defensive strategies used by gut commensals to counteract gut colonization by pathogens. The overall data collected from studies involving animal, in vitro, and in silico models are reported throughout the text and summarized in Table 1. Studies involving humans are not included in the table but are widely discussed in the appropriate subsections. The names of bacterial taxa in the lists are presented in descending order from the highest to the lowest taxonomical rank and in alphabetical order within the same rank, regardless of the relevance of increases or reductions in microbial abundances.

### 3.1. Clostridioides difficile

*Clostridioides difficile*, formerly known as *Clostridium difficile*, is a spore-forming, obligate anaerobic, Gram-positive bacterium [10]. It naturally colonizes the human intestinal tract after the ingestion of spores and inhabits the gut as a peaceful commensal. Percentages of *C. difficile* asymptomatic colonization range from 0% to 51% in the population, mainly depending on age, geography, access to healthcare structures and hospitalization, and other environmental factors [59,60,61,62]. However, some toxigenic strains are sadly known for their ability to cause antibiotic-associated diarrhea in hospitalized patients (healthcare-associated infections), taking advantage of the intestinal dysbiosis resulting from prolonged use of broad-spectrum antibiotics [63,64]. Nowadays, a high number of *C. difficile* infections (CDIs) are also acquired outside of hospitals (community-acquired infections). In fact, a recent report by the Centers for Disease Control and Prevention (CDC) declared that a total of 13,348 cases of CDI, consisting of 6769 community-acquired infections and 6579 healthcare-associated infections, occurred in the US in 2021 [65]. In addition to exposure to oral antibiotics, age (≥65 years), comorbidities (i.e., intestinal bowel disease, obesity, kidney diseases), gastric bypass, and concomitant therapies (i.e., chemotherapy, protonic pump inhibitor therapy) are all factors enhancing the risk of acquiring the infection [66]. Clinical symptoms of CDI range from mild/moderate diarrhea to life-threatening diseases (i.e., pseudomembranous colitis, fulminant colitis, toxic megacolon) [63] and mainly depend on the virulence of the infecting strain. For example, the hypervirulent *C. difficile* ribotype 027 strain displays high expression of toxins and antibiotic resistance, thus generally causing more severe infections [10].

The overgrowth of *C. difficile* in the intestinal tract is normally kept under control by gut commensals through the competition for nutrients and adhesion sites and the production of microbial-derived compounds, such as bacteriocins, SCFAs, and secondary bile acids [63,67]. Therefore, disruption of the gut microbiota is critical for CDI development [68], while the restoration of homeostatic bacterial diversity and abundance of the gut consortia is important for recovery [69]. The disruptive effects on the gut microbiota composition of vancomycin and fidaxomicin as standard CDI antibiotic treatments were widely evaluated in humans [70,71,72,73,74]. Orally administered vancomycin was shown to cause a marked reduction in *Actinomycetota* (i.e., *Bifidobacteriaceae*, *Choriobacteriaceae*), *Bacillota* (i.e., *Clostridiaceae*, *Eubacteriaceae*, *Lachnospiraceae*, *Ruminococcaceae*), and *Bacteroidota* (i.e., *Bacteroidaceae*, *Prevotellaceae*) and an increase in *Pseudomonadota* and *Lactobacillaceae* [72,73], thus resulting in aberrant microbial populations. Critical reductions in both bacterial biodiversity and total load were also observed in concomitance with the vancomycin-based treatment [74]. Surprisingly, fidaxomicin induced fewer variations and no increase in *Pseudomonadota* [70,71], acting as a reliable therapeutic solution considering the poor impact of this antibiotic on the intestinal communities. Similar to antibiotics, the role of fecal microbiota transplantation (FMT) and probiotics in facilitating successful recovery from CDI has also been extensively investigated [64,68].

Several clinical trials have been carried out to explore alterations in the fecal microbiota in patients suffering from CDI [67,68,69,75,76,77,78,79]. The majority of studies examining the bidirectional interaction between *C. difficile* and gut-residing microorganisms were conducted on human subjects, with animal and in vitro models being comparatively underused until now. The totality of clinical trials agreed in concluding that CDI is actually associated with an overall loss of α-diversity and marked gut dysbiosis. A recent comprehensive review by Vasilescu and colleagues reported that CDI in adults correlated with a dramatic reduction in *Actinomycetota*, *Bacillota*, *Bacteroidota*, *Bacteroidaceae*, *Bifidobacteriaceae* (i.e., *B. adolescentis*, *B*. *longum*), *Clostridiaceae* (i.e., *C. scindens*), *Lachnospiraceae*, *Ruminococcaceae*, *Alistipes*, *Anaerostipes*, *Bacteroides* (i.e., *B. ovatus*, *B. vulgatus*), *Blautia*, *Dorea*, *Ezakiella*, *Faecalibacterium*, *Megamonas*, *Odoribacter*, *Prevotella*, *Pseudobutyrivibrio*, *Roseburia*, *Streptococcus*, *Subdoligranulum*, and *Oscillibacter massiliensis*, as well as with a significant increase in *Pseudomonadota*, *Enterobacteriaceae*, *Enterobacter*, *Enterococcus*, *Finegoldia*, *Fusobacterium*, *Lactobacillus*, *Mycobacterium*, *Parabacteroides*, *Akkermansia muciniphila*, and *E. coli* [79]. On the other hand, in newborns, CDI was associated with a reduction in *Bacillota*, *Bacteroidota*, *Bifidobacterium*, and *Ruminococcus* and higher abundances of *Citrobacter*, *Enterococcus*, *Klebsiella*, *Shigella*, *E. coli*, and *Staphylococcus aureus* [79]. *Bacteroidota* and *A. muciniphila* showed the most controversial behavior in CDI since some studies reported their increase and others their reduction in the same condition [78].

To make the clinical issue even more complex, patients who have “resolved” CDI often suffer from *C. difficile* recurrences 2–8 weeks after the primary infection [68]. The gut microbiota of patients with recurrent CDIs was demonstrated to be even more dysbiotic than that of CDI patients, with lower α-diversity and levels of *Bacillota* and *Bacteroidota* [80,81]. The reason why recurrences of CDI occurred was investigated by Henson and co-authors, who hypothesized in silico the putative mechanisms at the basis of recurrent CDI [82]. The authors developed a computational model of gut microbiota in CDI, which revealed an overall reduction in the anabolism of secondary bile acids and an increase in the catabolism of aromatic amino acids. These in silico predictions suggested that the metabolism of expanding *Enterobacteriaceae* may help in creating a favorable intestinal environment for *C. difficile* spore germination, vegetative cell replication, and toxin synthesis [82].

Within the spectrum of animal models, mice have been mainly used to study CDI in relation to the gut microbiota. In mice, CDI correlated with higher abundances of *Akkermansia*, *Anaerotignum*, *Bacteroides*, *Clostridium*, *Enterocloster*, *Murimonas*, and *Turicibacter* [32]. Murine models were also useful in revealing that certain microbial communities in the gut could potentiate the severity of developed CDI [32]. In this study, germ-free mice were initially colonized by FMT from different human donors and then infected with *C. difficile* ribotype 027, thus developing CDIs of different severity on the basis of the transferred gut microbiota. In particular, mice harboring bacterial populations with a prevalence of *Enterococcus*, *Helicobacter*, and *Klebsiella* developed a more severe CDI in comparison to mice colonized by *Anaerotignum*, *Blautia*, *Lactonifactor*, and *Monoglobus* [32]. Therefore, infection severity and subsequent clinical manifestations strongly depend on the composition of the bacterial consortia residing in the gut. The susceptibility to CDI was also assessed in mice considering the administration of different dietary regimes [33]. For instance, a soybean-protein-based diet was demonstrated to increase intestinal levels of amino acids and protein derivates, promote murine gut colonization by *C. difficile*, and reduce survival rate after CDI compared to a regular purified diet [33]. The abundances of *Lactobacillus* spp. and *Ligilactobacillus murinus* increased, thus resulting in a higher genesis of extracellular amino acids facilitating *C. difficile* growth [33].

In vitro models have also been utilized for this purpose. Horvat and colleagues tested the pathogenic role of three different toxigenic *C. difficile* ribotypes (both vegetative cells and culture supernatants, separately; 027, 078, and 176) against in vitro-maintained fecal microbiota from children [34]. Abundances of *Bacillota*, *Bacteroidota*, and *Pseudomonadota* and overall α-diversity were found to be reduced for all the applied strains. In particular, vegetative cells and conditioned media of all ribotypes significantly reduced the levels of *Veillonella* and increased those of *Bacteroides* and *Clostridium* XIVa, with ribotypes 027 and 176 further inducing the specific lowering of *Lachnospiraceae* and *Ruminococcaceae*. Vegetative cells alone determined higher levels of *Morganella* and lower levels of *Flavonifractor*, whereas conditioned media surprisingly behaved in the opposite manner, reducing *Morganella* and expanding *Flavonifractor* [34]. As a result of these changes in microbial composition, metabolic profiles of in vitro-cultured microbiota also changed, positively influencing the sporulation process of *C. difficile* [34]. The effect of specific dietary compounds on *C. difficile* proliferation was also evaluated in an in vitro model mimicking CDI, named CDi-Screen [35]. Vegetative cells and spores of *C. difficile* ATCC 43599 were separately co-cultured with the gut microbiota in the model in the presence of 2′-fucosyllactose (2′-FL), which was shown to significantly inhibit the overgrowth of *C. difficile* in vitro, reduce its abundance, and enhance the levels of *Blautia* in a dose-dependent manner [35].

### 3.2. Campylobacter jejuni

*Campylobacter jejuni* is a slim, spiral-shaped, Gram-negative bacterium recognized as one of the most common foodborne pathogens in the world, especially in developed countries [83,84]. According to the recent 2021 zoonoses report of the European Food Safety Authority (EFSA), campylobacteriosis ranks first among foodborne gastrointestinal infections in Europe, with a total of 127,840 notified cases in 2021 alone [85]. The food vehicle often implicated in the transmission of zoonotic campylobacteriosis to humans is contaminated poultry meat [84]. In fact, it is very common for chickens, turkeys, and other avian species to asymptomatically harbor in their intestine a large number of *C. jejuni*, which can accidentally cross-contaminate meats intended for human consumption [86]. Campylobacteriosis in humans is typically associated with acute intestinal symptoms (i.e., diarrhea, abdominal cramps, hemorrhagic colitis, appendicitis), but sometimes long-term gastrointestinal pathologies (i.e., intestinal bowel disease, colorectal cancer, Barrett’s esophagus) or even extra-intestinal dissemination (i.e., bacteriemia and sepsis, endocarditis, septic thrombophlebitis, meningitis, brain abscesses, demyelinating neuropathies, Guillame-Barré syndrome, pneumonia) can occur [87].

The relationship between *C. jejuni* and the gut microbiota in humans has been rarely studied. In a pioneering study, Dicksved, Kampmann, and coworkers demonstrated that the fecal microbiota from humans naturally infected by *C. jejuni* displayed lower biodiversity compared to healthy individuals, although abundances of *Bacteroides*, *Escherichia*, *Phascolarctobacterium*, and *Streptococcus* were significantly increased [88,89]. Furthermore, *Dorea* and *Coprococcus* spp. residing in the gut, both belonging to *Lachnospiraceae* family, were pointed out as important actors in the protection against *C. jejuni* intestinal colonization in humans [89].

Most of the studies aimed at exploring *C. jejuni* pathogenicity and its involvement in human health were carried out in broiler chickens since poultry is the natural reservoir of *C. jejuni*. In particular, the role of poultry intestinal communities in colonization resistance was thoroughly investigated [90,91]. Colonization by *C. jejuni* in 56-day-old broiler chickens was associated with a reduction in *Corynebacterium* and *Lactobacillus* and an increase in *Ruminococcaceae* and *Streptococcus* [36]. The authors also found out that colonization was positively correlated with *Alistipes*, *Bacteroides*, *Blautia*, *Clostridium*, *Enterobacter*, *Enterococcus*, *Escherichia*-*Shigella*, *Faecalibacterium*, and *Gallibacterium* [36]. A more recent study based on *C. jejuni*-infected chickens showed that α-diversity and richness of gut consortia were higher than in healthy chickens and that several alterations emerged in the microbiota composition in concomitance with the *C. jejuni* colonization [37]. In particular, levels of *Barnesiella*, *Helicobacter*, *Methanocorpusculum*, *Parasutterella*, and *Rikenella* were increased, whereas *Eggerthellaceae*, *Lachnospiraceae*, *Clostridium*, *Lactobacillus*, *Monoglobus*, and *Parabacteroides* were significantly decreased [37]. Moreover, supplementation with probiotic *Bifidobacterium* and *Lactobacillus* spp. to poultry reduced the *C. jejuni* colonization rate [92,93,94,95], probably because of their positive in vitro-confirmed effects in promoting *C. jejuni* elimination and enhancing the expression of interleukins and co-stimulatory molecules [96]. The generation of secondary bile acids, such as deoxycholic acid, by probiotics and gut commensals was also recognized as a contributing factor capable of positively reshaping the intestinal microbiota and reducing *C. jejuni* counts in bird feces [97].

Mice have rarely been used as *C. jejuni* infection models, since the murine gut microbiota is intrinsically protective against intestinal colonization by *C. jejuni* [98,99]. In fact, while untreated mice were highly resistant to the foodborne infection by *C. jejuni* F38011, the oral administration of ampicillin, leading to profound alterations in the gut communities, resulted in increased intestinal colonization by *C. jejuni*. This alteration correlated with heightened symptoms, as well as with the extraintestinal spread of the bacterium to mesenteric lymph nodes and spleen [100]. The colonization resistance seemed to be associated with high abundances of *Bifidobacterium*, *Butyricicoccus*, *Clostridium* XI, *Coprobacillus*, *Hydrogenoanaerobacterium*, *Lactobacillus*, *Oscillibacter*, and *Roseburia*, while susceptibility to *C. jejuni* infection correlated with a prevalence of other clostridia and *Enterococcus* [36]. Moreover, the innate toll-like receptor 4 (TLR-4) response to *C. jejuni* lipooligosaccharide (LOS) is markedly weaker in mice than in humans [99]. For many years, these findings dissuaded researchers from developing suitable murine *C. jejuni* infection models. However, novel murine models were recently developed, thus opening the way for studies concerning *C. jejuni* in mammals [99]. In particular, genetically modified knockout mice for the single-Ig IL-1-related receptor (SIGIRR; *Sigirr*^−/−^) [101,102] or IL-10 (*IL-10*^−/−^) [103,104,105] were made available to sensitize mice to *C. jejuni* LOS. *Sigirr*^−/−^ or *IL-10*^−/−^ mice treated with oral broad-spectrum antibiotics or human FMT to disrupt the murine microbiota can represent a suitable mammal model for mimicking human *C. jejuni* infection, thus overcoming the disadvantages associated with the use of wild-type mice.

To date, no in vitro models have been developed and validated for investigating the influence of the human gut microbiota on *C. jejuni* colonization and infectious process or the effects of *C. jejuni* on the resident microbial communities.

### 3.3. Diarrheagenic Escherichia coli

*Escherichia coli* is a rod-shaped, genetically and metabolically versatile, Gram-negative bacterium [106]. The species includes strains that behave as commensals in the intestines of humans and other animals and opportunistic/pathogenic strains able to cause infection [106]. Among the pathogenic strains, uropathogenic *E. coli* (UPEC), sepsis-causing *E. coli* (SEPEC), and neonatal meningitis-associated *E. coli* (NMEC) are the three pathotypes determining extraintestinal infections (i.e., of the genitourinary tract, bloodstream, and central nervous system, respectively) in humans [107]. On the other hand, Shiga-toxin-producing *E. coli* (STEC), enterohemorrhagic *E. coli* (EHEC), enteropathogenic *E. coli* (EPEC), enteroaggregative *E. coli* (EAEC), enteroinvasive *E. coli* (EIEC), enterotoxigenic *E. coli* (ETEC), adherent-invasive *E. coli* (AIEC), diffusely adhering *E. coli* (DAEC), and cell-detaching *E. coli* (CDEC) are the nine pathotypes currently recognized as causative agents of *E. coli*-related human gastroenteritis [107]. Each pathotype displays its own virulence profile that allows the microorganism to establish the infectious process [108]. The ingestion of food or water contaminated with fecal material from infected individuals is the main route of infection [106], which mainly involves children (<5 years old), dysbiotic adults, and immunocompromised individuals in both developed and developing countries [109]. The most relevant worldwide *E. coli* foodborne outbreaks from 2006 to 2015 were reviewed by Yang and coworkers [109]. This study pointed out the main pathotypes involved in human disease in recent decades [109]. In the US, mainly in northwestern states, 6034 cases of STEC infection occurred in 2017, with STEC O157:H7 accounting for 40% of cases [110]. EFSA also reported the number and distribution of infections by STEC in the European territories in 2021, surprisingly showing a total of 6084 notified cases, of which 275 were foodborne cases from 31 separate outbreaks [85].

A robust healthy gut microbiota plays a crucial role in preventing diarrheagenic *E. coli* infections. Especially *Bacteroides* (i.e., *B*. *fragilis*, *B. thetaiotaomicron*) [111,112], *Lactobacillus* (i.e., *L. acidophilus*, *L. reuteri*) [113,114], *Bifidobacterium* (i.e., *B. breve*) [115,116], and butyrate-producing bacteria (i.e., *Clostridium butyricum* and *tyrobutyricum*, *Anaerostipes butyraticus*) [117,118,119] were shown able to reduce infection susceptibility in mice and cattle and protect animals from pathogenic *E. coli* colonization in different ways, including the restriction of bacterial growth and the inhibition of virulence gene expression [120]. In particular, among the various mechanisms of colonization resistance, the direct production of SCFAs (i.e., acetate), secondary bile acids, and bacteriocins by microbial commensals, as well as of antimicrobial peptides and secretory IgA by host eukaryotic cells, were shown to be the most effective means to counteract ETEC invasion [121]. Conversely, some commensals (i.e., *B. thetaiotaomicron*) make nutrients indirectly available for *E. coli* by degradation of complex polysaccharides or mucus layer and enhance the expression of *E. coli* virulence genes, thus promoting infection [120,122].

Apart from the pathogenic role of Shiga toxins as proinflammatory factors and harmful effectors inducing cell death and disruption of microvilli of the intestinal epithelial layer, limited information is available regarding the interaction between STEC and bacterial consortia residing in the gut. The impact of STEC, particularly of strain O26:H11, on the gut microbiota was evaluated by Gigliucci and coworkers in naturally infected Italian children [123]. Infected children displayed lower levels of *Bifidobacteriales*, *Clostridiales*, *Bifidobacterium*, *Butyrivibrio*, *Coprococcus*, *Faecalibacterium*, and *Roseburia* in their fecal samples compared to healthy controls, as well as a higher abundance of *Lactobacillus* [123]. Curiously, strain O157:H7, which is the main cause of global infections by STEC, has never been investigated for this purpose in vivo or in vitro. Gallardo and colleagues also explored the effect of diarrheagenic *E. coli* on the gut communities of Chilean children [124]. After infection, the infant gut microbiota underwent severe alterations, including a reduction in *Bacillota* and the expansion of *Bacteroidota*, *Pseudomonadota*, *Enterobacteriaceae*, *Bacteroides*, *Escherichia*-*Shigella*, *Pseudocitrobacter*, *Escherichia albertii*, *Citrobacter werkmanii*, *Haemophilus sputorum*, and *Yersinia enterocolitica* subspecies *paleartica* [124]. Comparable results were obtained by Mizutani, in a study that also highlighted a reduced α-diversity in the microbial consortia of infected individuals [125]. Lately, the pathways of histamine and L-ornithine metabolism were found to be altered in association with gut microbiota changes after *E. coli* infection [126]. Histamine was shown to be overproduced and linked to an increased amount of resident *Bifidobacterium stercoris*, *Citrobacter werkmanii*, *Enterobacter hormaechei*, and *Shigella* spp. Conversely, L-ornithine exhibited an opposing pattern, being less prevalent in conjunction with a higher prevalence of *Enterococcus faecalis*, *Escherichia* spp., and *Streptococcus anginosus* [126]. High levels of ETEC in fecal samples from infected children and adults from Bangladesh were found to be associated with a greater probability of co-infection with other pathogenic *E. coli* strains (i.e., EAEC) [127]. Additionally, they displayed a higher prevalence of antimicrobial resistance genes, and distinctive alterations in the gut microbiota were observed depending on the age of the individuals [127]. In children, the observed alterations were limited to a decrease in *Bifidobacterium* and an increase in *Enterobacteriaceae*, *Streptococcus*, and *Comamonas*. In contrast, adults exhibited more pronounced scenarios of ETEC-associated gut dysbiosis, characterized by increased abundances of *Campylobacteria*, *Gammaproteobacteria*, *Burkholderiales*, *Enterobacteriaceae*, *Pasteurellaceae*, *Veillonellaceae*, *Citrobacter*, *Klebsiella*, and *Salmonella enterica* and a reduction in the abundance of the *Bacilli* and *Clostridia* classes [127].

To overcome the challenges posed by the limited susceptibility of mice to natural and experimental infection by EHEC and EPEC, potential approaches include the oral administration of broad-spectrum antibiotics to disrupt the balance of intestinal microbiota or the utilization of germ-free mice [122]. Stromberg and colleagues used germ-free mice colonized with altered Schaedler flora (ASF), a synthetic community composed of eight standard bacterial species [128], and infected them with EHEC 278F2 [129]. ASF mice were effectively colonized and infected with EHEC, providing a well-defined murine model suitable for conducting infection experiments with pathogenic *E*. *coli* [129]. However, mice subjected to such treatment are useless if the aim of the study is to explore the impact of pathogenic *E. coli* on the murine gut microbiota and, in a translational context, on that of humans.

As regards ETEC, two in vitro models mimicking the intestinal mucosa were developed to selectively decipher the *E. coli* interactions with cultured mucus-adhering microbes [22,38]. The colonic mucus-associated microbiota of piglets was cultured in the MPigut-IVM system in the presence of the ETEC strain Ec105 [38]. The authors demonstrated substantial deviations in the examined communities, revealing a significant increase in *Bacillota*, *Bacteroidota*, *Enterococcaceae*, *Prevotellaceae*, *Eisenbergiella*, *Enterococcus*, *Morganella*, *Peptoniphilus*, and *Tyzzerella* along with a notable decrease in *Actinomycetota*, *Acidaminococcaceae*, *Bacteroidiaceae*, *Erysipelotrichaceae*, *Ruminococcaceae*, and *Veillonellaceae*. Contextually, higher levels of propionate, 3-phenylpropionate, caproate, valerate, isovalerate, and tyramine, and higher expressions of pro-inflammatory factors encoding genes were detected when ETEC was present in the model [38]. Sauvaitre and colleagues tested the ETEC strain H10407 in the TIM-1 system simulating the upper human intestinal tract [22]. As a result, the α-diversity of the cultured consortia decreased after infection, and a reduced abundance of *Bifidobacterium*, *Clostridium*, and *Lactobacillus* was observed. Conversely, members of the *Roseburia* genus were significantly expanded [22].

### 3.4. Non-Typhoidal Salmonella enterica Serovars Typhimurium and Enteritidis

Microorganisms belonging to the *Salmonella* genus are rod-shaped, facultative anaerobic, Gram-negative bacteria that cause the second most frequently reported foodborne infection in humans after campylobacteriosis [130]. Since *Salmonella* spp. are abundant in the intestinal tract of several animal species, which represent their natural reservoir, foods of animal origin, vegetables, and water contaminated with feces are the main vehicles for gastrointestinal infections in humans [131]. Depending on the serovars, *S. enterica* is able to cause both typhoidal (i.e., *S. enterica* serovars Typhi and Paratyphi) and non-typhoidal (i.e., *S. enterica* serovars Typhimurium and Enteritidis) salmonellosis in humans. While the typhoidal clinical picture usually includes enteric typhoid/paratyphoid fever, bacteriemia, and gastroenteritis, non-typhoidal manifestations are mainly associated with only foodborne gastroenteritis [130]. In Europe, a total of 60,050 cases of salmonellosis were reported in 2021, including 6755 cases resulting from 733 separate foodborne outbreaks [85]. *S. enterica* Enteritidis, Typhimurium, and Infantis were, in order, the most isolated serovars determining non-typhoidal salmonellosis [85]. Furthermore, the number of infections due to multi-drug resistant *Salmonella* strains is increasing worldwide [132].

As for other intestinal pathogens, gut commensals act as the first line of defense to resist the colonization of the intestinal mucosa by *Salmonella* [133]. The competition for adhesion sites and nutrients, the production of bacteriocins, antimicrobial peptides, and SCFAs, and the stimulation of mucosal secretory IgA are the main anti-*Salmonella* mechanisms deployed by the gut microbiota and the intestinal epithelium to prevent colonization [134,135,136]. The active production of propionate by *Bacteroides* spp. was shown to directly inhibit *S*. *ent*. Typhimurium growth in vitro [135]. Furthermore, the intestinal commensalism by colicin- and/or microcin-producing *Enterobacteriaceae* reduced the colonization rate by *S. enterica* [136]. It is important to emphasize that certain strains of *Salmonella* are also able to secrete colicin and microcins, which can target and eliminate intestinal microbes, particularly those belonging to *Enterobacteriaceae* and Gram-negative bacteria. This underlines the existence of a competitive intra-family struggle for dominance in the intestinal environment. However, since *Salmonella* displays a limited repertoire of bacteriocins compared to *E. coli* and other *Enterobacteriaceae*, alternative mechanisms must be activated to escape colonization resistance and infiltrate the intestinal mucosa. These mechanisms include the use of the type VI secretion system (T6SS) and injected effector proteins to eliminate the local microbiota [136]. By using an in vitro two-compartment co-culture system, *S*. *ent*. Typhimurium SL1344 growth was shown to be inhibited by *E. coli*, confirming the abovementioned struggle among *Enterobacteriaceae* [137]. Conversely, the survival of *Lactobacillus gasseri* and *Bifidobacterium bifidum* was strongly reduced by *Salmonella ent*. Typhimurium, indicating that *Salmonella* not only causes harm to human tissues but also directly affects resident commensal bacteria [137].

Several animal models, such as mice, pigs, and chickens, have been used to explore the dynamic interactions between *Salmonella enterica* and the gut microbial consortia [138,139]. Initially, mice were proposed as a model to study *S*. *ent*. Typhimurium pathogenicity, interaction with and impact on the gut microbiota during the infectious process, and both local and systemic *Salmonella*-related diseases [134]. *S*. *ent*. Typhimurium caused a reduction in α-diversity of the intestinal microbial consortia and an increase in *Enterobacteriaceae*, *Enterobacter cancerogenus*, *Escherichia fergusonii*, and *Proteus penneri* in mice [39]. On the other hand, real-time quantitative PCR experiments showed that the same species induced a strong reduction in the total bacterial load within the intestine, along with reductions in *Enterococcus*, *Lactobacillus*, *Clostridium coccoides*, and *Eubacterium rectale* compared to uninfected mice [40].

Unlike *S*. *ent*. Choleraesuis, which has evolved to specifically infect swine hosts, *S*. *ent*. Typhimurium accidentally infects pigs, but they were still exploited as reliable *Salmonella* infection models [41]. While weaned pigs infected by *S*. *ent*. Typhimurium displayed higher abundances of *Lactobacillus* and *Oscillaspira* than uninfected controls, *Ruminococcaceae*, *Coprococcus*, *Lachnospira*, *Prevotella*, and *Ruminococcus* were more abundant in uninfected swine [41]. Despite these important alterations in the gut microbial composition, no differences in Shannon indexes between the two groups were reported. Moreover, post-weaning microbiota maturation and abundances of commensals correlated with breastfeeding were revealed to be determining factors for pig susceptibility to *S*. *ent*. Typhimurium infection [41]. Another study in swine models reported that *S*. *ent*. Typhimurium increased the intestinal levels of *Anaerobacter*, *Barnesiella*, *Catenibacterium*, *Pediococcus*, *Prevotella*, *Pseudobutyrivibrio*, *Sporacetigenium*, *Turicibacter*, and *Xylanibacter* [42]. Regarding the mucus-adhering microbiota in pigs, there was an increase in *Citrobacter* levels in the presence of *S*. *ent*. Typhimurium, whereas levels of *Bifidobacterium*, *Clostridium*, *Lactobacillus*, and *Ruminococcus* significantly decreased [43].

Chickens were also extensively exploited as *S*. *ent*. Typhimurium and, mainly, Enteritidis infection models. Robinson and colleagues infected broiler chickens with *S*. *ent*. Typhimurium ATCC 14028 to evaluate if the subsequent modulation of the cecal microbiota could facilitate *Salmonella* colonization [44]. The tested pathogenic strain determined an overall reduction in species richness associated with a time-dependent lowering of *Bacillaceae*, *Escherichia*, and *Lactobacillus*, as well as an increase in *Bacteroides*, thus allowing more effective and stable colonization of chicken cecum by *S*. *ent*. Typhimurium [44]. Several studies investigated the impact of *S*. *ent*. Enteritidis on chicken gut microbiota, providing complementary results [45,46,47,48]. At the order level, infection with *S*. *ent*. Enteritidis 147 was associated with an increase in *Enterobacteriales* and a reduction in *Bifidobacteriales*, *Clostridiales*, and *Lactobacillales* [45]. At the family level, Mon and coworkers initially reported that *S*. *ent*. Enteritidis TN2 infection induced an expansion of the *Enterobacteriaceae* family, of which *Salmonella* is a member, and a reduction in *Lachnospiraceae* in young chicks [46]. Subsequently, the same research group obtained further comprehensive insights into the impact of the TN2 strain on the chicken microbiota [47]. Chao-1 indexes were significantly different between uninfected and infected chickens, with the latter displaying an increased α-diversity. Abundances of *Bacillaceae*, *Eubacteriaceae*, *Peptostreptococcaceae*, *Ruminococcaceae*, and *Streptococcaceae* were increased after *Salmonella* infection, while the opposite occurred for *Anaeroplasmataceae*, *Chromatiaceae*, *Lactobacillaceae*, *Leuconostocaceae*, *Planococcaceae*, *Rhizobiaceae*, and *Turicibacteriaceae* [47]. Considering the main genera, *Anaerostipes*, *Anaerotruncus*, *Bacillus*, *Enterococcus*, *Flavonifractor*, and *Intestinimonas* were increased by *S*. *ent*. Enteritidis CVCC3377, whereas *Blautia* and *Shuttleworthia* were reduced [48]. The overall results obtained via animal infection models could be helpful to partially comprehend the role of the human gut microbiota in protecting from *S. enterica* infection and the pathogen’s negative impact on intestinal homeostasis and systemic health.

A few in vitro models have been developed to study *Salmonella*–host “microbiota” interactions, but none of these models actually includes the entire gut microbiota up to date. They rather harbor eukaryotic cells in co-culture with single microbial strains or primordial synthetic communities [140]. For this reason, the analysis of those works, despite being interesting, goes beyond the aim of the present review and will not be included herein.

### 3.5. Shigella flexneri and Shigella sonnei

*Shigella flexneri* and *Shigella sonnei* are Gram-negative bacteria responsible for almost the totality of cases of shigellosis in the world and are common causes of traveler’s diarrhea in developing countries [141,142]. Shigellosis is a highly transmittable infection acquired by the ingestion of food and water contaminated with at least 10 shigellas or through a direct fecal–oral route, and mainly affects children (<5 years old) [142]. Shigellas can colonize the crypts of the colonic mucosa despite the presence of resident microbiota and invade the epithelium leading to its disruption [49,143]. Clinical manifestations of shigellosis include watery/mucoid/bloody diarrhea, gastrointestinal discomforts, nausea, vomiting, abdominal cramps, and fever [144]. According to a 2023 CDC report, approximately 5% of *Shigella* infections in 2022 were caused by extensively multi-drug resistant strains compared to a net 0% in 2015 [145]. In fact, *Shigella* spp., especially *S. sonnei*, can easily acquire antibiotic resistance genes through horizontal genic transfer, thus leading to the expansion of resistant strains worldwide [49].

Considering their remarkably low infectious doses, *S. flexneri* and *S. sonnei* are presumed to have developed specific defensive mechanisms to survive in the intestinal environment and triumph in the competition against the local microbiota while being definitely outnumbered [49]. It is probable that the production of bacteriocins, such as SF1 and colicin, by certain strains of *S. flexneri* and *S. sonnei*, respectively, may help in killing members of the gut microbiota (i.e., *E. coli*, *Bacteroides fragilis*), thus ensuring a selective advantage for the pathogen during the colonization and invasion processes [146,147]. On the other hand, bacteriocins produced by intestinal commensals were demonstrated to be equally effective in protecting against *S. flexneri* colonization. In particular, colicin produced by *E. coli* was shown to be protective in *E. coli*-monocolonized germ-free mice and guinea pigs, thus allowing us to infer possible competition among different members of the *Enterobacteriaceae* family [49]. Species of *Lactobacillus* were also pointed out as important defenders against *Shigella* infection, due to their anti-inflammatory activity and surface proteins of the S-layer that inhibit the adhesion of *S. sonnei* to epithelial cells [148,149,150,151]. *Lactobacillus reuteri*, *L. ruminis*, *L.* DJF-RP24, *L.* KLDS 1.0718, and *L.* TSK G32.2 were specifically demonstrated to carry out antagonistic interactions against *Shigella* [151,152].

Children naturally infected with *Shigella* have been commonly recruited to study the microbial dynamics arising from the interaction between *Shigella* spp. and the infant gut microbiota [152,153]. Lindsay and colleagues demonstrated that infant diarrheal stool containing high levels of *Shigella* displayed lower abundances of *Prevotella* and higher abundances of *Streptococcus* compared to healthy controls [153]. Contextually, Ndungo and co-authors pointed out that there were no differences in α-diversity between healthy and infected children and that *Fusicatenibacter saccharivorans* and *Lachnospiraceae* NK4A136 were significantly increased after *Shigella* infection [152].

With regard to studies on animal models, *Shigella flexneri* ATCC 12022 was orally and intraperitoneally administered to mice to evaluate its impact on the murine gut microbiota [50]. Intraperitoneal inoculation led to decreased α-diversity without severely altering the composition of intestinal consortia. In contrast, oral administration resulted in a significant decrease in *Lactobacillaceae*, *Alistipes*, and *Lactobacillus*, as well as a strong increase in *Lachnospiraceae*, *Muribaculaceae*, *Prevotellaceae*, *Alloprevotella*, and *Prevotella* [50]. The increase in *Prevotella* and *Alloprevotella* was hypothesized to be associated with more massive inflammatory states and recruitment of inflammatory cells in the intestine. The reduction in *Lactobacillaceae* and *Lactobacillus* could correlate with lower resistance to colonization and more serious *Shigella* infection. In addition to being more severe, changes in the gut microbiota caused by the natural oral route of infection were also faster compared to those obtained via the intraperitoneal route [50].

*S. flexneri* and *S. sonnei* have never been tested together with the gut microbiota within in vitro models. As for other enteropathogens, the in vitro approach to evaluate how *Shigella* spp. act in the intestine and interact with the human gut microbiota could be clinically relevant.

### 3.6. Vibrio cholerae

*Vibrio cholerae* is a curved, rod-shaped, Gram-negative bacterium, globally known as the causative agent of cholera, an acute watery diarrhea illness that has epidemically been affecting humans for centuries [154]. *V. cholerae* is endemic in many regions of Africa and Asia and a recent report by the World Health Organization (WHO) stated that a total of 472,697 cases of cholera were reported worldwide in 2022 [155]. Gastrointestinal infection with *V. cholerae* is commonly acquired through the ingestion of contaminated food and water [154].

Once it reaches the intestine, *V. cholerae* must adapt to the intestinal environment, penetrate within the mucus layer by hydrolyzing mucins, and adhere to the intestinal epithelium for stable colonization [156]. There, *V. cholerae* can release the cholera toxin and deliver effector proteins to eukaryotic and prokaryotic cells through its T6SS, which were pointed out as two crucial virulence factors of the pathogen [157]. Especially T6SS was demonstrated to have a role in killing gut commensals and shaping the host gut microbiota. In fact, *V. cholerae* T6SS mediated the killing of *E. coli* and other intestinal Gram-negatives in vitro [157], suggesting that *V. cholerae* acts directly against intestinal microorganisms, thus worsening the outcome of the infectious process. On the other hand, microbes of the gut microbiota can suppress the expression of *V. cholerae* T6SS by converting bile salts to their deconjugated forms via microbial bile salt hydrolases [158,159]. Although the interaction between deconjugated bile salts and T6SS-encoding genes is totally unknown, bile salts play a key role in preventing infection by *V. cholerae.* Qin and coworkers also demonstrated that taurocholate was able to disrupt the mature biofilm of *V. cholerae* by altering its matrix and promoting its degradation [158], further corroborating the anti-*Vibrio* effects of certain bile acids. However, the crosstalk between intestinal microbes and *V. cholerae* is very complex and many other factors surely contribute to that intricate interaction (i.e., SCFAs, bacteriocins, quorum sensing) [158,160].

Since members of the *Vibrio* genus naturally live in aquatic environments, including freshwaters, estuarine waters, and sea waters [156], fishes were proposed to be suitable models for studying *V. cholerae* pathogenicity [51,161,162]. In particular, zebrafish (*Danio rerio*) are endemic in those areas where *V. cholerae* is also endemic and their immune system displays high similarity with that of humans [163]. Notably, their intestinal microbiota does not require alterations to allow *V. cholerae* colonization [51,164]. Breen and colleagues investigated the efficacy of five different strains of *V. cholerae* (i.e., 254-93, AM-19226, V52, E7946, and N16961) in determining infection in zebrafish, demonstrating that strain-specific qualitative and quantitative modulations of gut microbiota occurred during infection [51]. All strains determined a reduction in *Aeromonas*, *Cloacibacterium*, and *Fluviicola*, whereas differences were highlighted concerning the increase in specific taxa after infection with different strains. *V. cholerae* 254-93 raised levels of *Pseudomonas*, AM-19226 of *Plesiomonas* and *Novosphingobium*, V52 of *Cetobacterium* and *Plesiomonas*, E7946 of *Plesiomonas* and *Enterobacteriaceae*, and N16961 of *Fictibacillus*. Moreover, while V52 and E7946 determined an increase in α-diversity and total bacterial load in zebrafish gut communities, AM-19226 and N16961 strains induced a reduction in diversity, with no quantitative alterations in the microbial load [51]. Altogether, the results obtained in zebrafish largely contributed to the knowledge of the effects of *V. cholerae* infection on intestinal microbial populations.

Other animal models were tested as *V. cholerae* infection models, although requiring physiological and/or surgical modifications and removal of their own intestinal microbiota to be suitable infection models [51]. Cholera toxin was shown to be as lethal for wild-type mice as for humans, but its mechanism of action does not determine watery diarrhea in mice, which is, conversely, the main symptom in humans [165]. The murine microbiota is highly resistant to *V. cholerae* colonization since mice are not natural hosts for the bacterium [165]. Nevertheless, investigations conducted in clindamycin-treated and germ-free mice showed the capacity of *Bacteroides* spp. and, in particular, *Bacteroides vulgatus*, a relevant commensal of both the murine and human gut microbiota, to suppress *V. cholerae* infection by reducing the pathogen intestinal count by 75-fold [166]. This finding suggested that certain *Bacteroides*-derived metabolites could be implied in the resistance against *V. cholerae*. Since mice infected by *V. cholerae* displayed lower levels of intestinal SCFAs (i.e., propionic acid, butyric acid, and valeric acid) and *B. vulgatus* is able to synthesize large amounts of propionic and butyric acids [167], it was hypothesized that SCFAs produced by *B. vulgatus* could act as main molecules involved in the *Bacteroides*-mediated antagonism against *V. cholerae* [158,166].

Currently, there are no established in vitro models to represent the effect of *V. cholerae* on the gut microbiota. However, a recent in silico model predicted the level of *V. cholerae* infection in humans based on the composition of gut communities [52]. The authors declared that putative high levels of *Bacteroides*, *Prevotella*, and *Ruminococcus* and low abundances of *Streptococcus* were associated with a resistant phenotype in humans. Interestingly, *Bacteroides* was pointed out again as a protective genus of the gut microbiota against *V. cholerae* infection, confirming the data obtained from mice models.

### 3.7. Bacillus cereus

*Bacillus cereus* is a rod-shaped, spore-forming, Gram-positive bacterium responsible for food poisonings (i.e., emetic and diarrheal syndromes) and severe extra-intestinal infections (i.e., bacteremia and sepsis, endophthalmitis, endocarditis, and infections of the central nervous system, respiratory system, genitourinary tract, wounds, and mammary glands) in humans and mammals [168,169,170,171]. The production of spores and the ability to form biofilms make *B. cereus* highly resistant and globally distributed in soil, water, organic debris, and the gastrointestinal tracts of many animal species, including humans. Although it is estimated that gastrointestinal infections by *B. cereus* are common, the number of reported worldwide foodborne infections is low and probably underestimated. This discrepancy may be attributed to scarce diagnoses correlated with the modest clinical relevance and the self-limiting nature of the gastrointestinal symptoms [172]. However, severe localized outbreaks have been registered in recent decades [170,173,174,175,176]. Conversely, some strains of *B. cereus* are totally harmless or even display beneficial properties. For this reason, they were characterized and made available for probiotic administration to animals [177,178].

Although the mechanisms involved in the pathogenesis of *B. cereus* infections are now well known, *B cereus* interactions with gut commensals are still almost unexplored. The effects modulating the gut microbiota after oral administration of pathogenic or probiotic strains of *B. cereus* were demonstrated in vivo in different animal models, including rodents, fishes, and insects. While the administration of spores and vegetative cells of *B. cereus* F4433/73R to rats did not cause substantial alterations in the gut community by PCR-DGGE, concomitant plate counts described a significant reduction in the total amount of coliforms, aerobes, and anaerobes [53]. In mice, the ingestion of the probiotic *B. cereus* strain HMPM18123 was demonstrated to ameliorate symptoms of dextran sulfate sodium (DSS)-induced colitis, by improving intestinal barrier integrity, reducing local inflammation and macrophage infiltration, and modulating the gut microbiota [54,179]. Microbial diversity was partially restored in *B. cereus*-treated mice, with higher abundances of *Bacillota*, *Verrucomicrobiota*, *Lachnospiraceae*, *Muribaculaceae*, *Rikenellaceae*, *Akkermansia*, *Jeotgalicoccus*, *Lactobacillus*, and *Roseburia*, and lower levels of *Pseudomonadota*, *Prevotellaceae*, and *Bacteroides* than in DSS-induced colitis models [54]. In Nile tilapia (*Oreochromis niloticus*) the addition of the probiotic *B. cereus* strain NY5 to aquaculture water determined an increase in intestinal *Peptostreptococcaceae*, *Clostridium*, and *Acetobacterium* and a reduction in *Pseudomonas* [55], thus revealing a propensity of *B. cereus* to induce the expansion of Gram-positive microbes in fishes. A similar result was also obtained in Pengze crucian carps (*Carassius auratus* var. Pengze) when *B. cereus* was administered to solve dysbiosis associated with a high-plant-protein diet [56]. *B. cereus*-treated fishes displayed improved growth performance and gut microbial diversity, with increasing levels of *Clostridium* and *Romboutsia* and a reduction in *Cetobacterium* [176]. The authors hypothesized that clostridia were pivotal in improving fish health after the high protein diet, considering their relevant contribution to amino acid, lipid, and carbohydrate metabolisms [56]. The diamondback moth (*Plutella xylostella*) was also investigated as a lepidopteran model for this purpose. *Bacillus cereus* ATCC 17788, unable to produce bacteriocins, and the zwittermicin A-producing *B. cereus* strain 6A4 were separately injected in *P. xylostella*, resulting in a significant reduction in *Enterobacter* spp. after the administration of both strains [57]. Moreover, Raymond and coworkers found out that the strain ATCC 17788 was not associated with overall alterations in microbial biodiversity in the insect gut, while strain 6A4 became the predominant bacterium within the microbial community profoundly altered by zwittermicin A [57]. These findings suggest that the ability of a bacterial strain to synthetize and actively secrete bacteriocins can be a determinant in accentuating differences in the outcomes even within the same animal model.

The impact of *B. cereus* on gut communities was also investigated in vitro by Calvigioni and colleagues [58]. Following a combined approach of 16S rDNA-targeting real-time qPCR and Illumina sequencing, microbial consortia of in vitro cultured fecal samples were analyzed after the addition of vegetative cells or culture supernatants of the pathogenic *B. cereus* strain ATCC 14579. The obtained results showed that *B. cereus* was able to reduce the total bacterial load, *Pseudomonadota*, *Akkermansia*, *Escherichia-Shigella*, *Faecalibacterium*, and *Lactobacillus* and increase the amount of *Bifidobacterium*, *Clostridium*, and *Mitsuokella* [58]. The increase in *Bifidobacterium* spp. in the presence of *B. cereus* could be explained by analogous findings obtained in previous studies on *Bacillus subtilis* C-3102, which was shown to be able to secrete specific bifidogenic factors (i.e., Val-based cyclic-dipeptides) [180,181]. However, the production of such bifidogenic factors has never been confirmed in *B. cereus* ATCC 14579.

## 4. Concluding Remarks and Perspectives

The present review aimed at exploring the intricate net of interactions between the most relevant enteropathogens and the gut microbiota, focusing on the alterations in microbial composition derived from the presence of the infectious agent. Infections caused by different enteropathogens lead to distinct outcomes not only with regard to the clinical manifestations but also to the modulation of the gut microbiota. These infections often affect the biodiversity and richness of the microbial populations, leading to alterations in the proportions of specific resident taxa. Pooling data from animal studies, in vitro models, and in silico predictions, it becomes evident that Gram-negative pathogens typically compete with other Gram-negative commensals for gut colonization. Conversely, Gram-positive pathogens tend to cooperate with other resident Gram-positive bacteria rather than engage in competition during the infection process, for example promoting the growth of commensal *Clostridium*, *Romboutsia*, or *Mitsuokella* spp. *Bifidobacterium*, *Lactobacillus*, and SCFA- and bacteriocin-producing commensals were confirmed to be protective against exogenous and endogenous enteric infections. Although several studies were cited in this review, there is a clear a dearth in the scientific literature of studies using in vitro systems for this purpose since only *C. difficile*, *E. coli*, and *B. cereus* have been investigated in vitro together with the gut microbiota. This fact demonstrates that researchers are still focusing their attention and efforts on human studies and animal models, rather than being committed to designing and developing new reliable in vitro models that may overcome the intrinsic limitations of humans and animals. In vitro models should now be recognized as novel powerful tools for their capacity to elucidate situations where animal models have limitations. These innovative models have the potential to provide insights into the mechanisms and the impact that enteropathogens have on the intestinal microbiota and vice versa. In the future, those findings, together with the knowledge derived from human and animal studies, could contribute to the global comprehension of the pathogenic role of enteric pathogens, not only in determining the damage to host cells and tissues but also in making the gut microbiota dysbiotic, thus amplifying the pathological outcome of the infection.

## Figures and Tables

**Table 1:** Summary of studies using animal, in vitro, and in silico models for gut microbiota research in association with infections by human enteropathogens.

Bacterial Species	Used Models	Results	References
*Clostridioides difficile*	Mice	↑ *Akkermansia*, *Anaerotignum*, *Bacteroides*, *Clostridium*, *Enterocloster*, *Murimonas*, *Turicibacter* Develop more severe CDI ^1^ when ↑ *Enterococcus*, *Helicobacter*, *Klebsiella*	[32]
	Mice	Soy-protein-based diet induces ↑ gut colonization by *C. difficile*, *Lactobacillus*, *Ligilactobacillus murinus* ↓ survival rate to CDI ^1^	[33]
	In vitro	↑ *Bacteroides*, *Clostridium* XIVa ↓ α-diversity, *Bacillota*, *Bacteroidota*, *Pseudomonadota*, *Lachnospiraceae*, *Ruminococcaceae*, *Veillonella*	[34]
	In vitro	When 2′-FL ^2^ was present ↑ *Blautia* ↓ *C. difficile*	[35]
*Campylobacter jejuni*	Broiler chickens	↑ *Ruminococcaceae*, *Streptococcus* ↓ *Corynebacterium*, *Lactobacillus*	[36]
	Broiler chickens	↑ α-diversity, *Barnesiella*, *Helicobacter*, *Methanocorpusculum*, *Parasutterella*, *Rikenella* ↓ *Eggerthellaceae*, *Lachnospiraceae*, *Clostridium*, *Lactobacillus*, *Monoglobus*, *Parabacteroides*	[37]
	Mice	Resistant to *C. jejuni* colonization when ↑ *Bifidobacterium*, *Butyricicoccus*, *Clostridium* XI, *Coprobacillus*, *Hydrogenoanaerobacterium*, *Lactobacillus*, *Oscillibacter*, *Roseburia* ↓ Other clostridia, *Enterococcus*	[36]
*Escherichia coli*	In vitro	↑ *Bacillota*, *Bacteroidota*, *Enterococcaceae*, *Prevotellaceae*, *Eisenbergiella*, *Enterococcus*, *Morganella*, *Peptoniphilus*, *Tyzzerella* ↓ *Actinomycetota*, *Acidaminococcaceae*, *Bacteroidiaceae*, *Erysipelotrichaceae*, *Ruminococcaceae*, *Veillonellaceae*	[38]
	In vitro	↑ *Roseburia* ↓ α-diversity, *Bifidobacterium*, *Clostridium*, *Lactobacillus*	[22]
*Salmonella enterica* serovars Typhimurium and Enteritidis	Mice (Typhimurium)	↑ *Enterobacteriaceae*, *Enterobacter cancerogenus*, *Escherichia fergusonii*, *Proteus penneri* ↓ α-diversity	[39]
	Mice (Typhimurium)	↓ Total bacterial load, *Enterococcus*, *Lactobacillus*, *Clostridium coccoides*, *Eubacterium rectale*	[40]
	Pigs (Typhimurium)	↑ *Lactobacillus*, *Oscillaspira* ↓ *Ruminococcaceae*, *Coprococcus*, *Lachnospira*, *Prevotella*, *Ruminococcus*	[41]
	Pigs (Typhimurium)	↑ *Anaerobacter*, *Barnesiella*, *Catenibacterium*, *Pediococcus*, *Prevotella*, *Pseudobutyrivibrio*, *Sporacetigenium*, *Turicibacter*, *Xylanibacter*	[42]
	Pigs (Typhimurium)	↑ *Citrobacter* ↓ *Bifidobacterium*, *Clostridium*, *Lactobacillus*, *Ruminococcus*	[43]
	Broiler chickens (Typhimurium)	↑ *Bacteroides* ↓ Species richness, *Bacillaceae*, *Escherichia*, *Lactobacillus*	[44]
	Broiler chickens (Enteritidis)	↑ *Enterobacteriales* ↓ *Bifidobacteriales*, *Clostridiales*, *Lactobacillales*	[45]
	Broiler chickens (Enteritidis)	↑ *Enterobacteriaceae* ↓ *Lachnospiraceae*	[46]
	Broiler chickens (Enteritidis)	↑ α-diversity, *Bacillaceae*, *Eubacteriaceae*, *Peptostreptococcaceae*, *Ruminococcaceae*, *Streptococcaceae* ↓ *Anaeroplasmataceae*, *Chromatiaceae*, *Lactobacillaceae*, *Leuconostocaceae*, *Planococcaceae*, *Rhizobiaceae*, *Turicibacteriaceae*	[47]
	Broiler chickens (Enteritidis)	↑ *Anaerostipes*, *Anaerotruncus*, *Bacillus*, *Enterococcus*, *Flavonifractor*, *Intestinimonas* ↓ *Blautia*, *Shuttleworthia*	[48]
*Shigella flexneri* *Shigella sonnei*	Mice (*S. flexneri*)	Resistant to *S. flexneri* colonization when ↑ colicin-producing *E. coli*	[49]
	Mice (*S. flexneri*)	↑ *Lachnospiraceae*, *Muribaculaceae*, *Prevotellaceae*, *Alloprevotella*, *Prevotella* ↓ *Lactobacillaceae*, *Alistipes*, *Lactobacillus*	[50]
*Vibrio cholerae*	Zebrafishes	↑ *Enterobacteriaceae*, *Cetobacterium*, *Fictibacillus*, *Novosphingobium*, *Plesiomonas*, *Pseudomonas* ↓ *Aeromonas*, *Cloacibacterium*, *Fluviicola*	[51]
	In silico	Resistant to *V. cholerae* colonization when ↑ *Bacteroides*, *Prevotella*, *Ruminococcus* ↓ *Streptococcus*	[52]
*Bacillus cereus*	Rats	↓ Coliforms, aerobes, anaerobes	[53]
	Mice	↑ *Bacillota*, *Verrucomicrobiota*, *Lachnospiraceae*, *Muribaculaceae*, *Rikenellaceae*, *Akkermansia*, *Jeotgalicoccus*, *Lactobacillus*, *Roseburia* ↓ *Pseudomonadota*, *Prevotellaceae*, *Bacteroides*	[54]
	Nile tilapias	↑ *Peptostreptococcaceae*, *Clostridium*, *Acetobacterium* ↓ *Pseudomonas*	[55]
	Pengze crucian carps	↑ Growth performance, α-diversity, *Clostridium*, *Romboutsia* ↓ *Cetobacterium*	[56]
	Diamondback moths	↓ *Enterobacter*	[57]
	In vitro	↑ *Bifidobacterium*, *Clostridium*, *Mitsuokella* ↓ Total bacterial load, *Pseudomonadota*, *Akkermansia*, *Escherichia*-*Shigella*, *Faecalibacterium*, *Lactobacillus*	[58]

Abbreviations: ^1^ CDI: *Clostridioides difficile* infection; ^2^ 2′-FL: 2′-fucosyllactose.

## Data Availability

Not applicable.
